# Supplementary figures and images for: Multifunction fluorescence open source in vivo/in vitro imaging system (openIVIS)
Source: PLoS One. 2024 Mar 18;19(3):e0299875. doi: 10.1371/journal.pone.0299875 (PMC10947658; doi:10.1371/journal.pone.0299875)

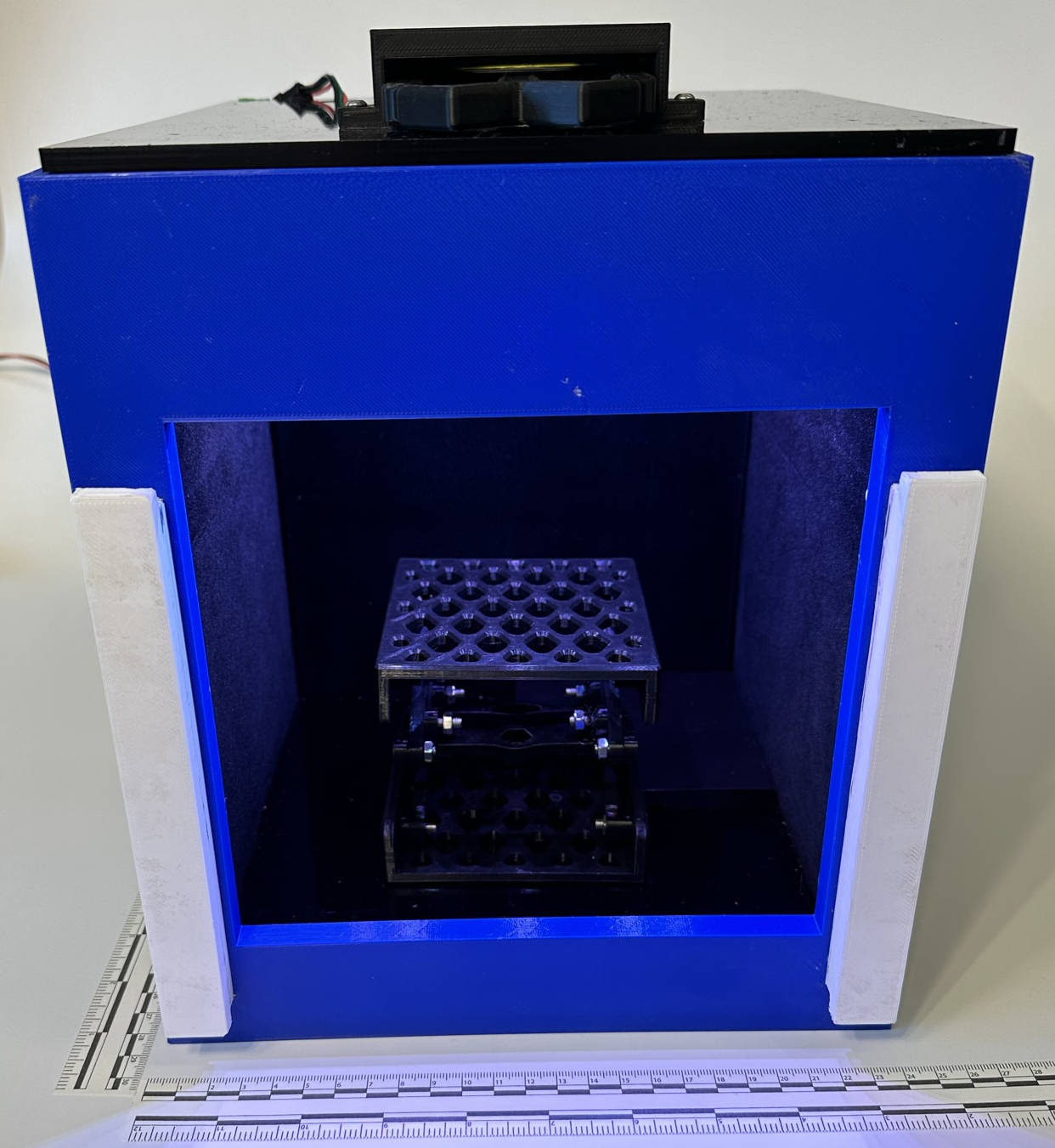

Supplement: S1 Fig — (TIF) [file pone.0299875.s001.tif]

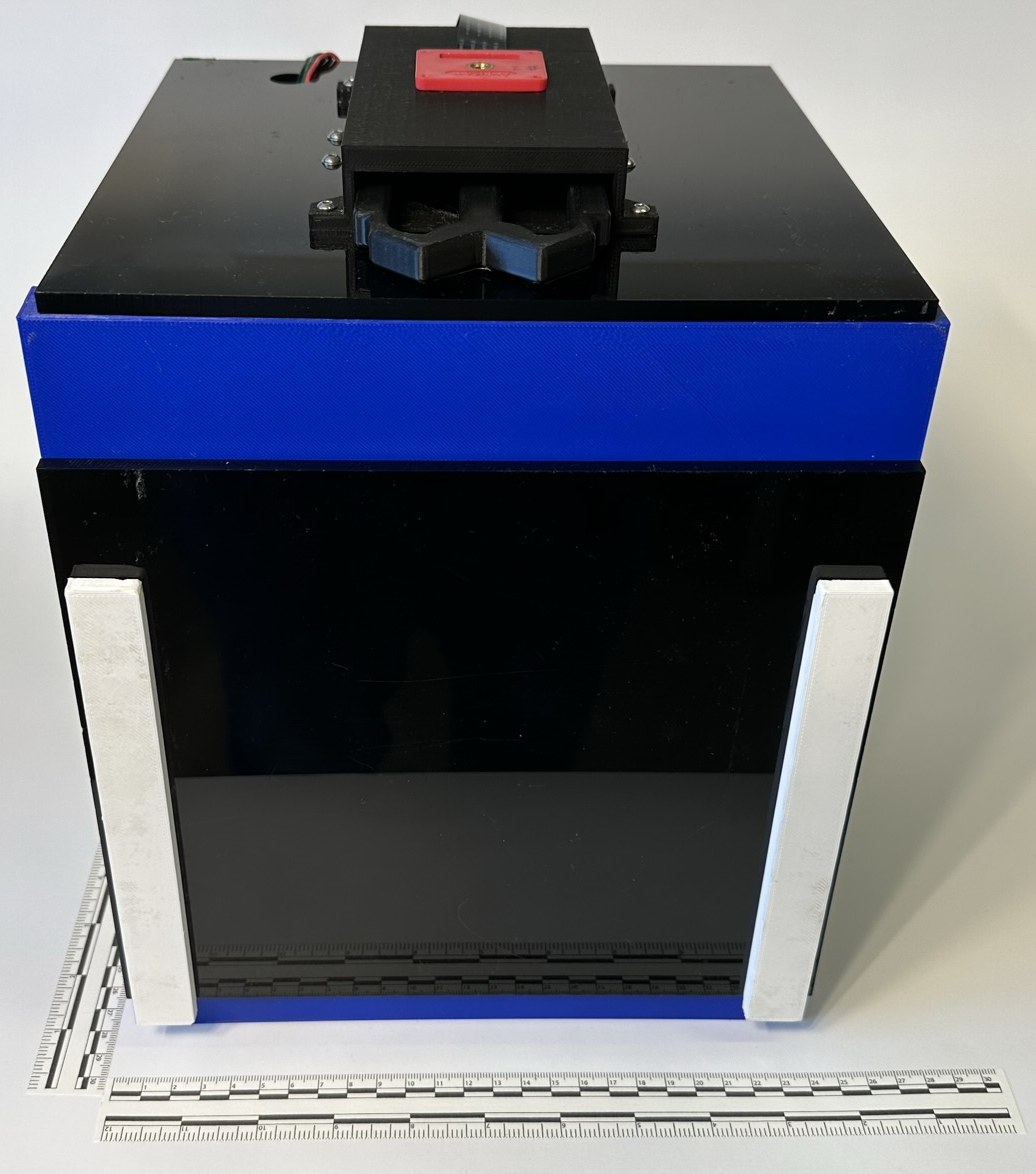

Supplement: S2 Fig — (TIF) [file pone.0299875.s002.tif]

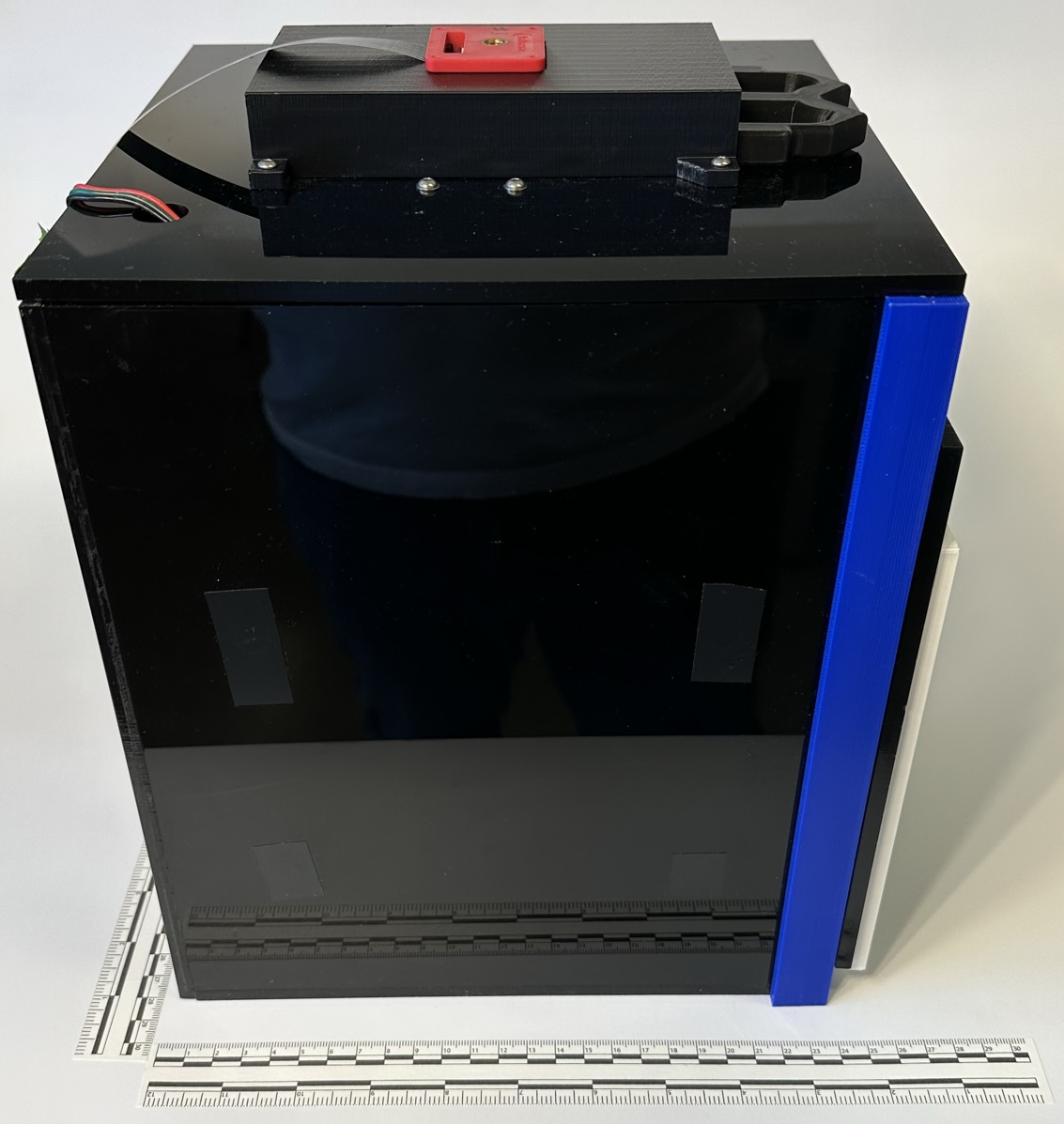

Supplement: S3 Fig — (TIF) [file pone.0299875.s003.tif]

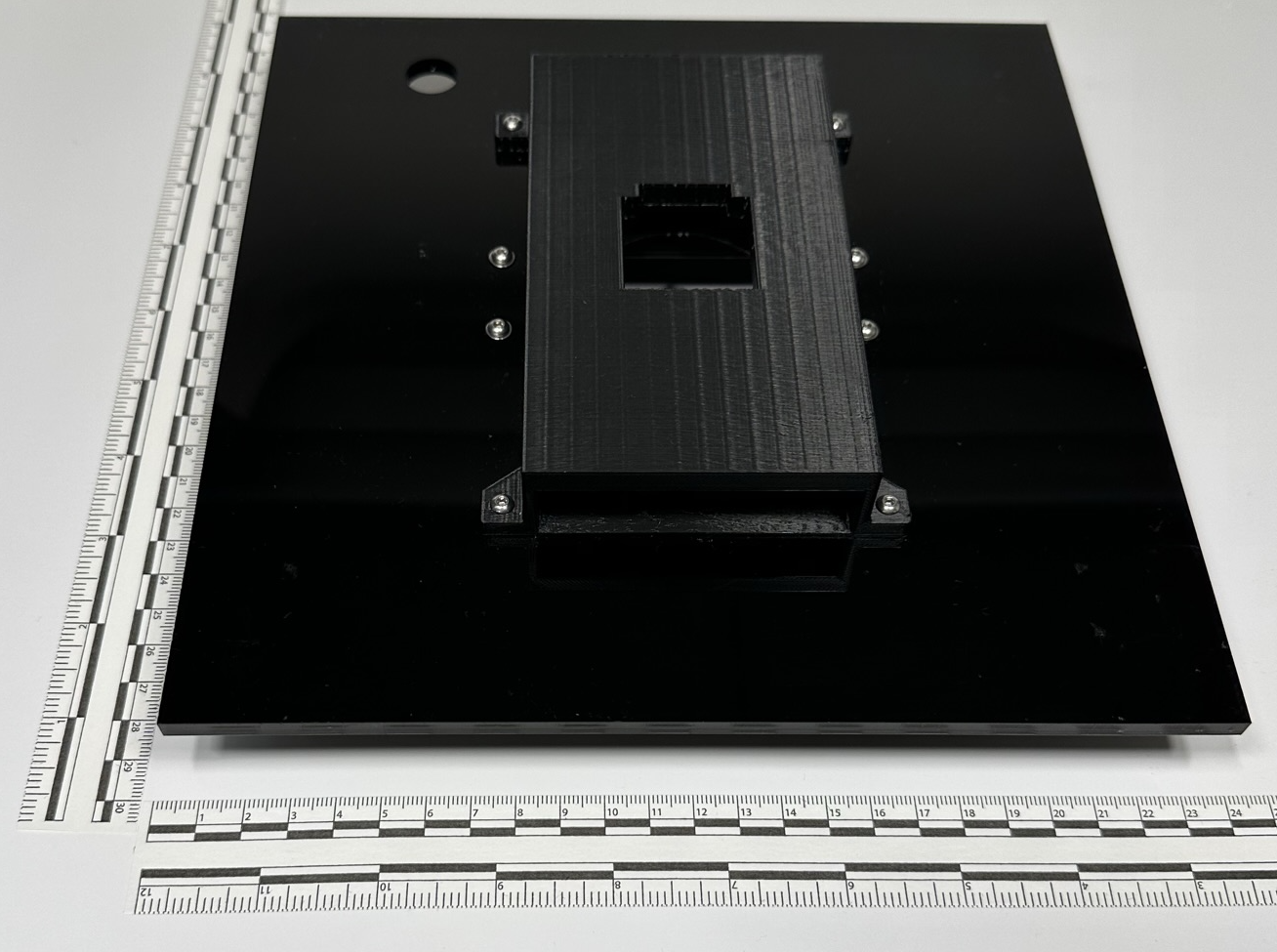

Supplement: S4 Fig — (TIF) [file pone.0299875.s004.tif]

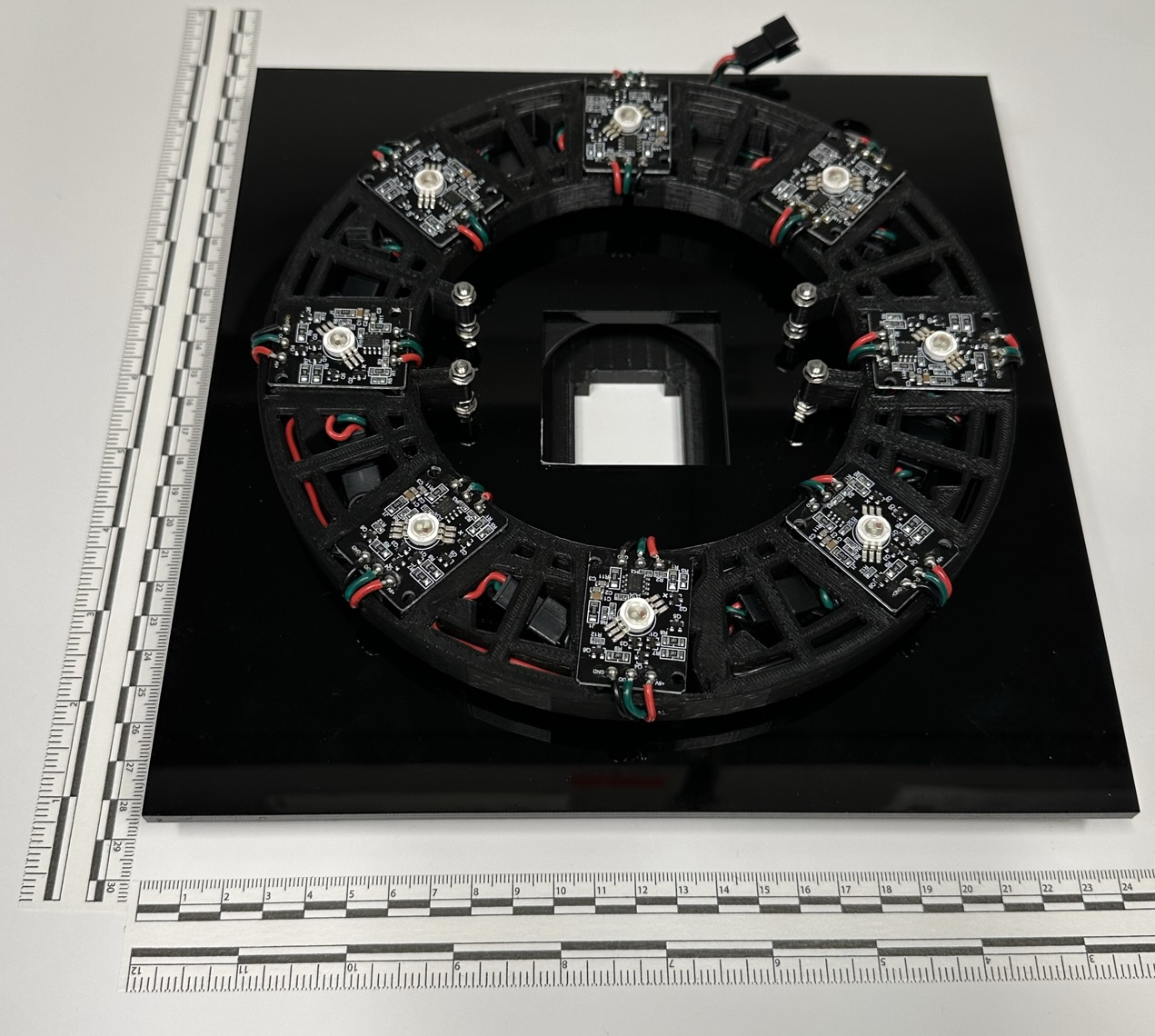

Supplement: S5 Fig — (TIF) [file pone.0299875.s005.tif]

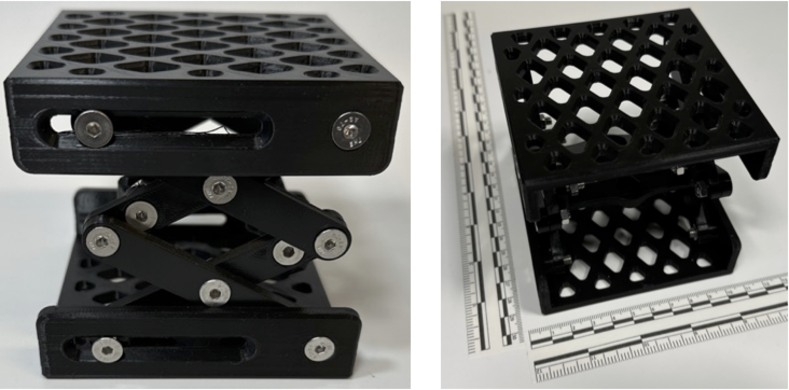

Supplement: S6 Fig — (TIF) [file pone.0299875.s006.tif]

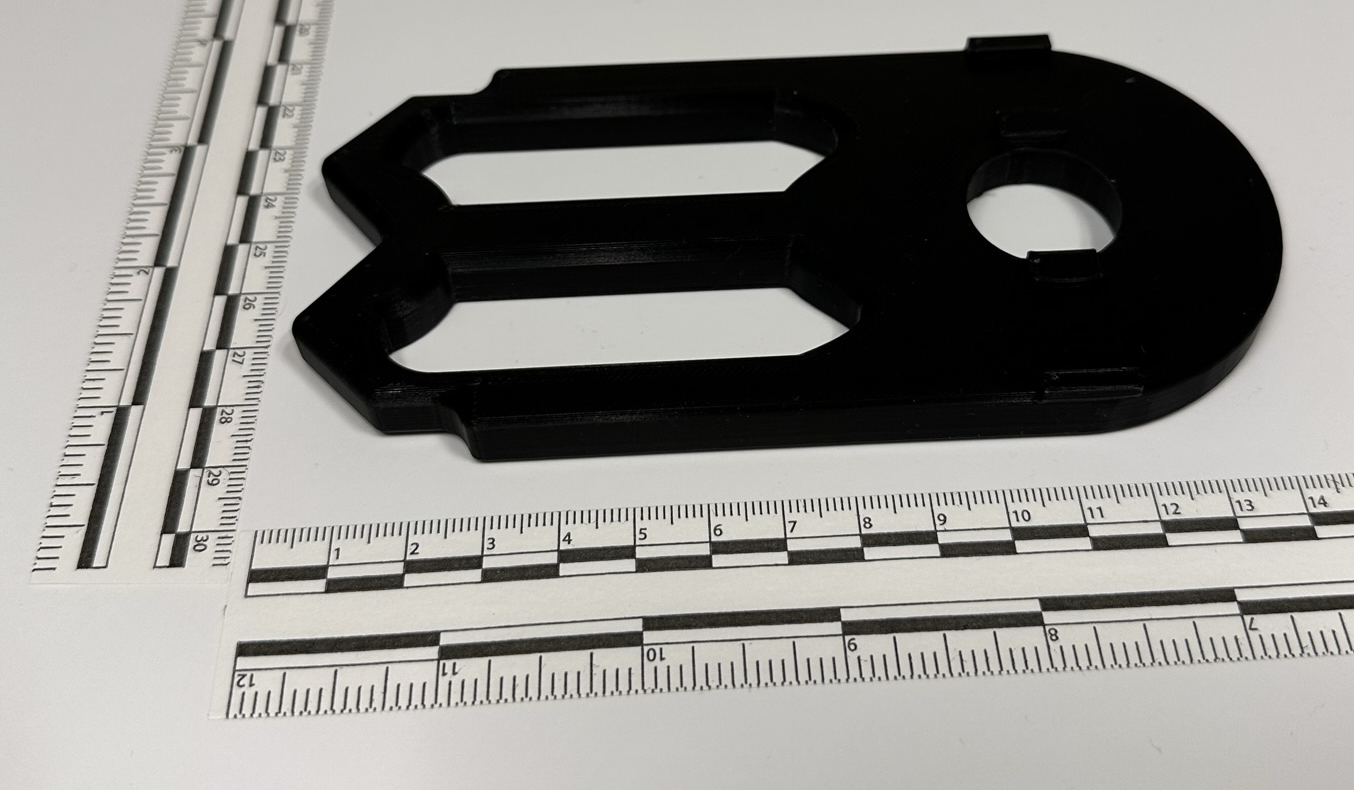

Supplement: S7 Fig — (TIF) [file pone.0299875.s007.tif]

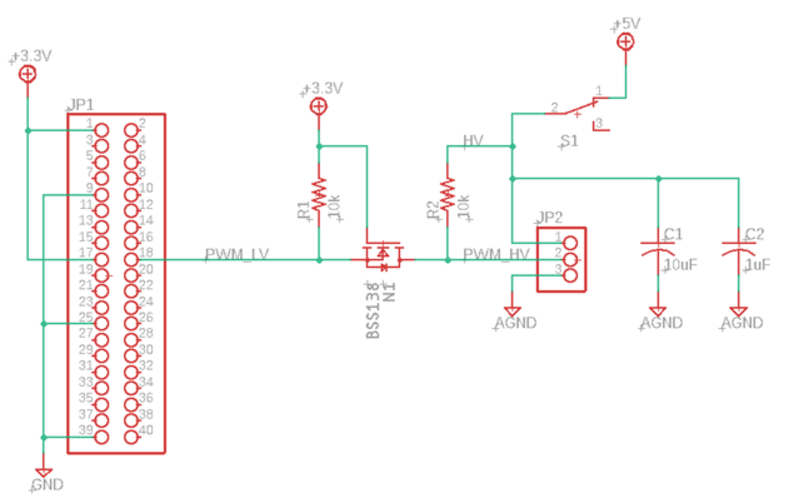

Supplement: S8 Fig — (TIF) [file pone.0299875.s008.tif]

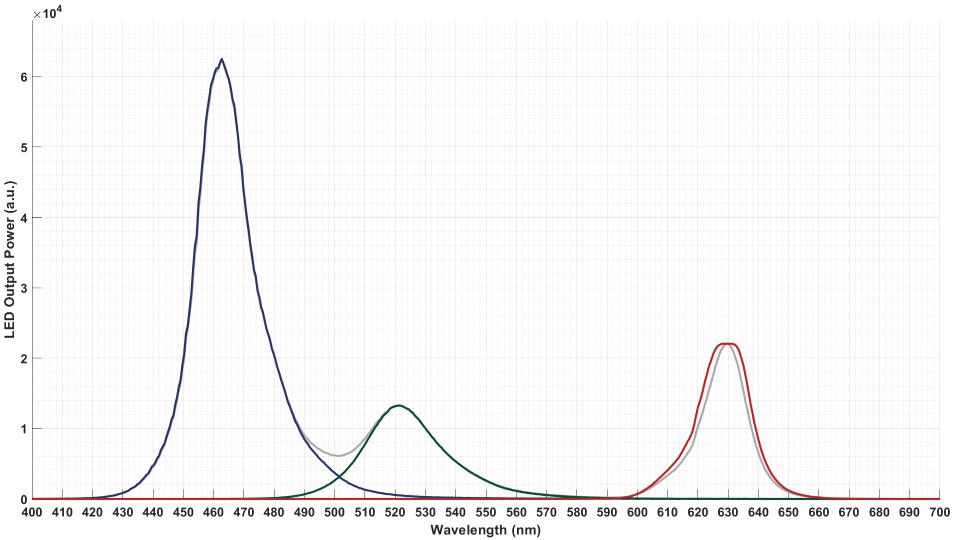

Supplement: S9 Fig — (TIF) [file pone.0299875.s009.tif]

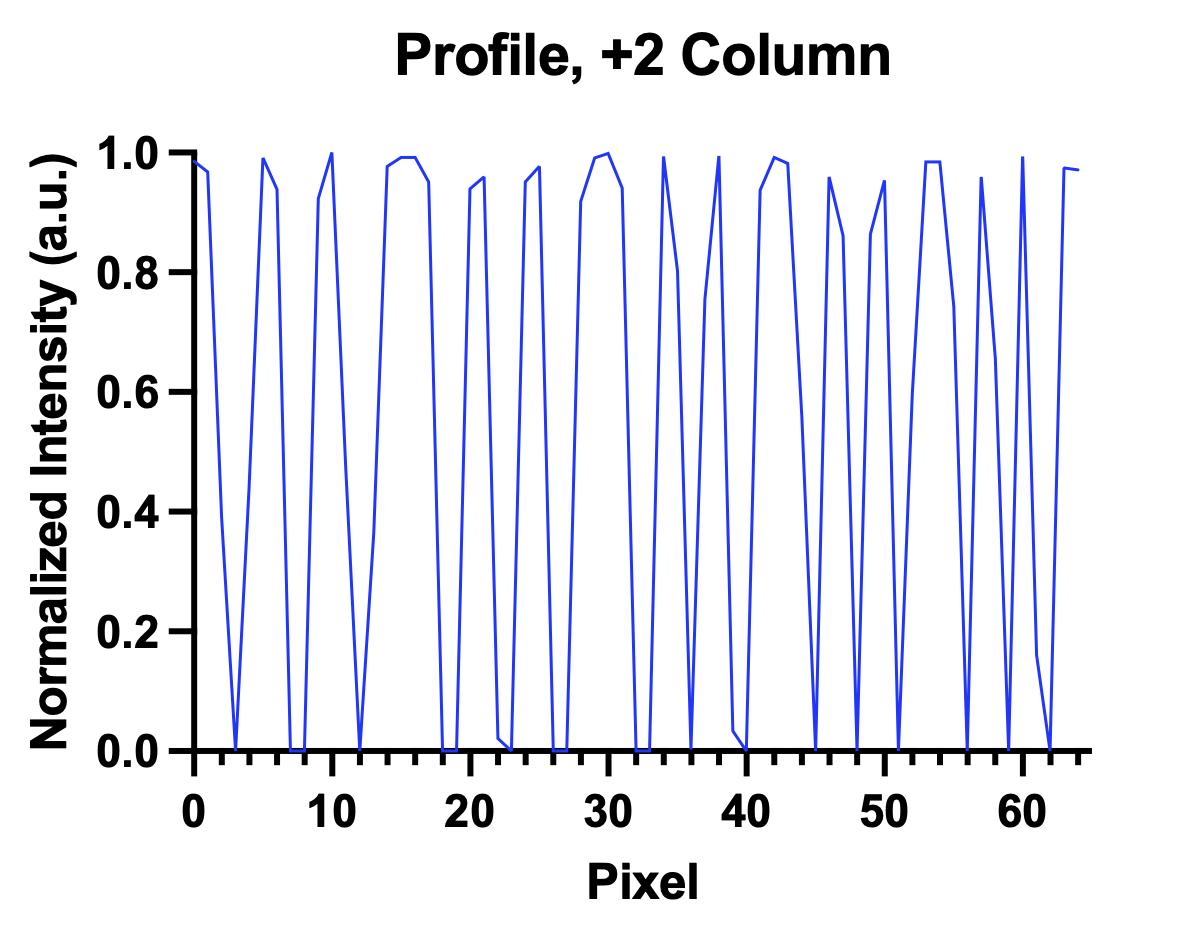

Supplement: S10 Fig — (TIF) [file pone.0299875.s010.tif]

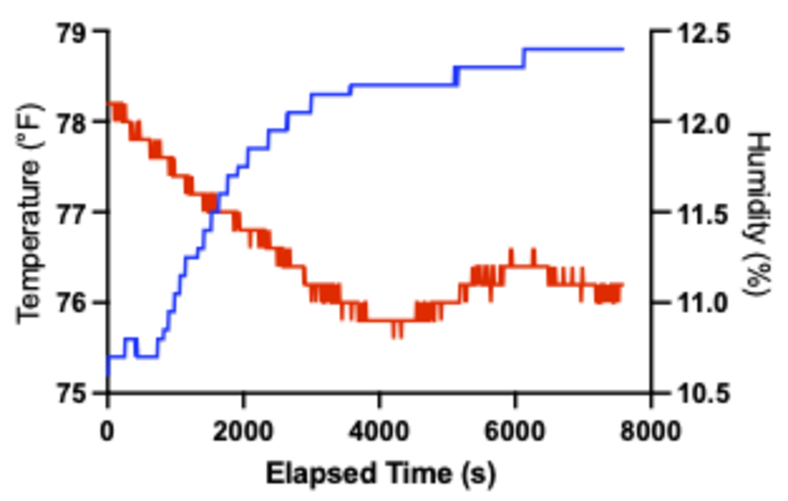

Supplement: S11 Fig — (TIF) [file pone.0299875.s011.tif]

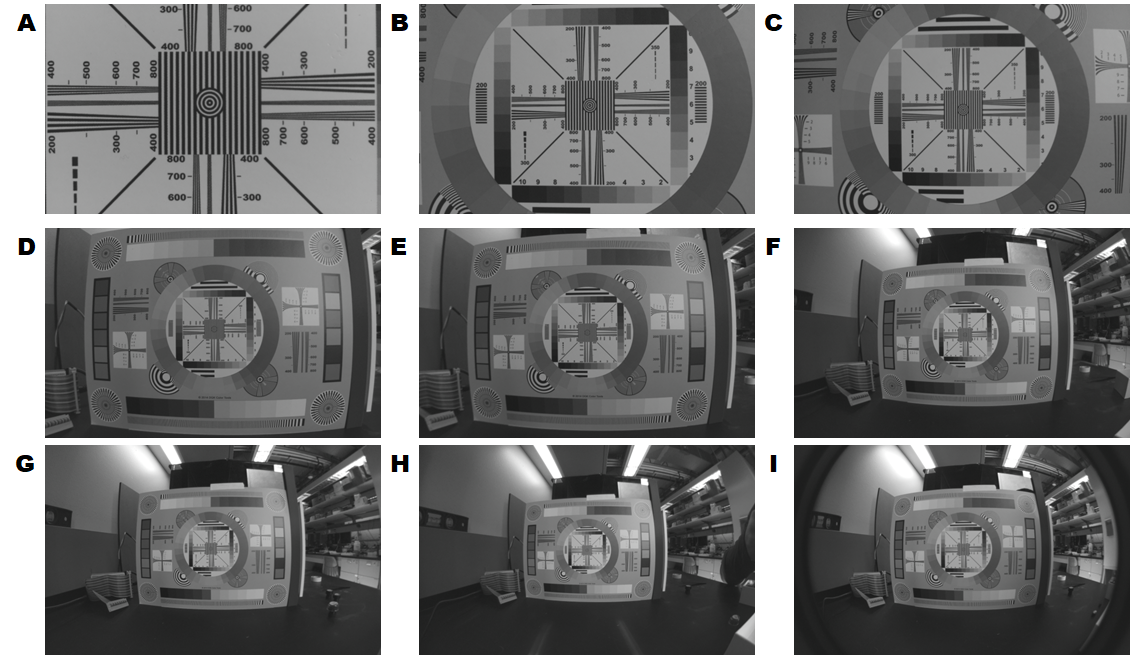

Supplement: S12 Fig — (A) 10° FOV (B) 20° FOV (C) 40° FOV (D) 60° FOV (E) 80° FOV (F) 100° FOV (G) 120° FOV (H) 140° FOV (I) 160° FOV. (TIF) [file pone.0299875.s012.tif]

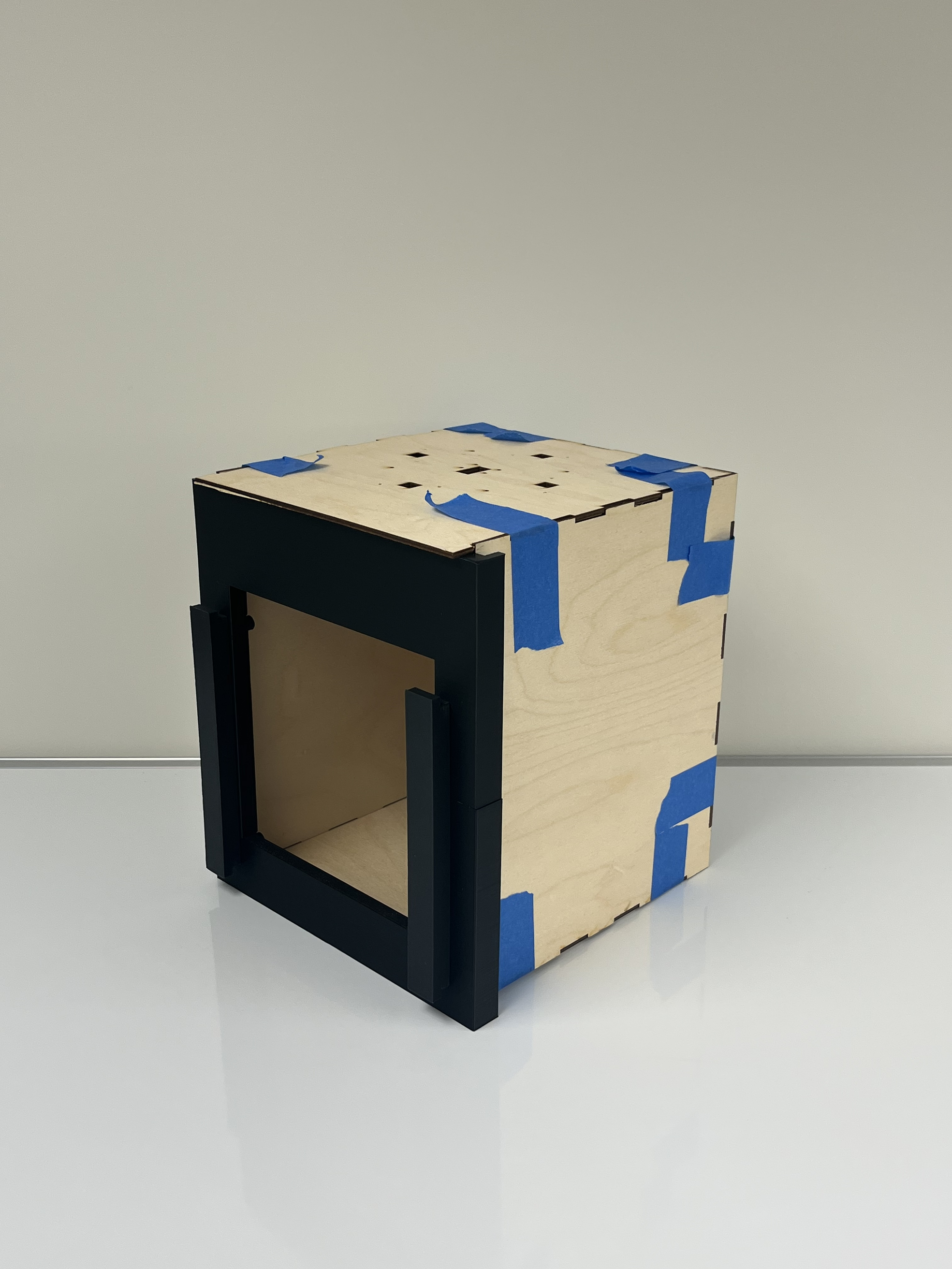

Supplement: S13 Fig — (TIF) [file pone.0299875.s013.tif]

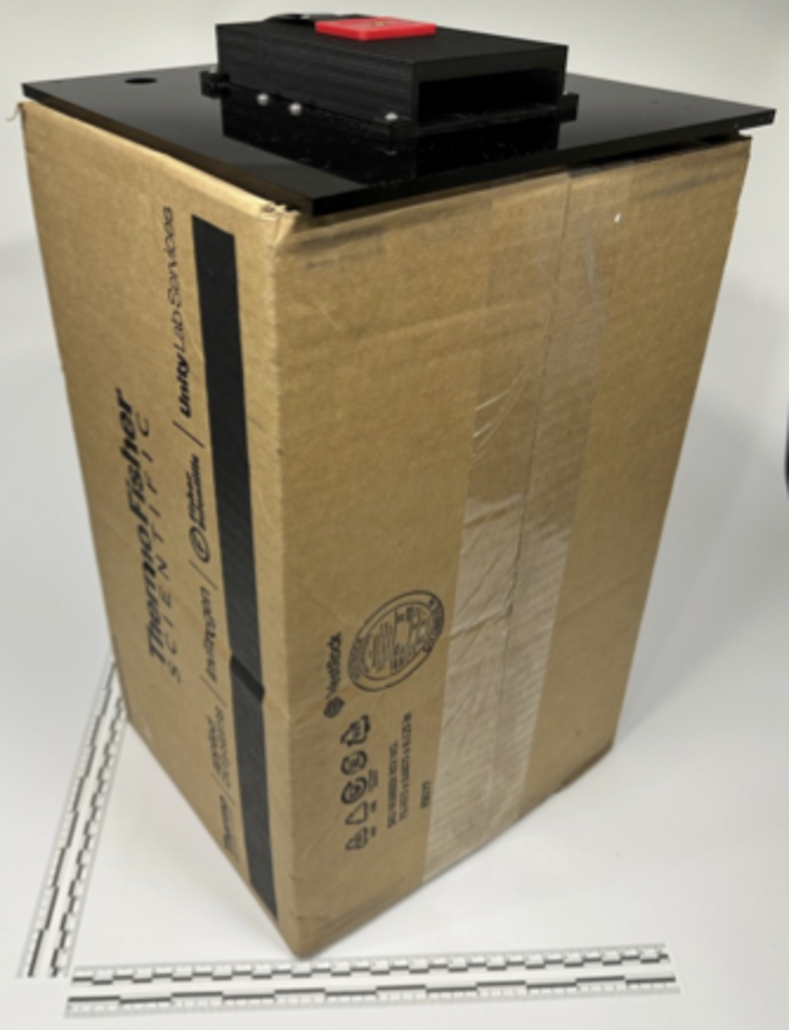

Supplement: S14 Fig — (TIF) [file pone.0299875.s014.tif]

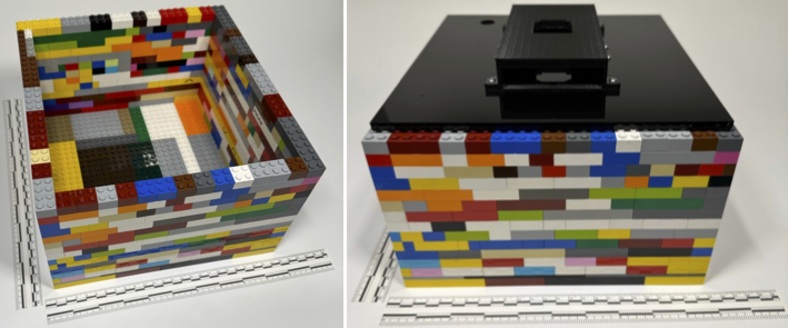

Supplement: S15 Fig — (TIF) [file pone.0299875.s015.tif]

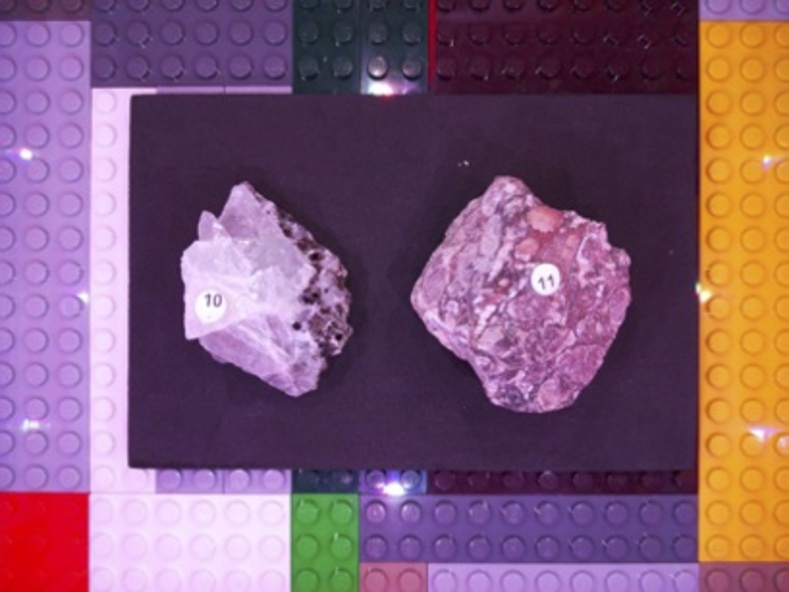

Supplement: S16 Fig — Left mineral is Fluorite from Utah, Right mineral is Turritella Agate from Wyoming. (TIF) [file pone.0299875.s016.tif]

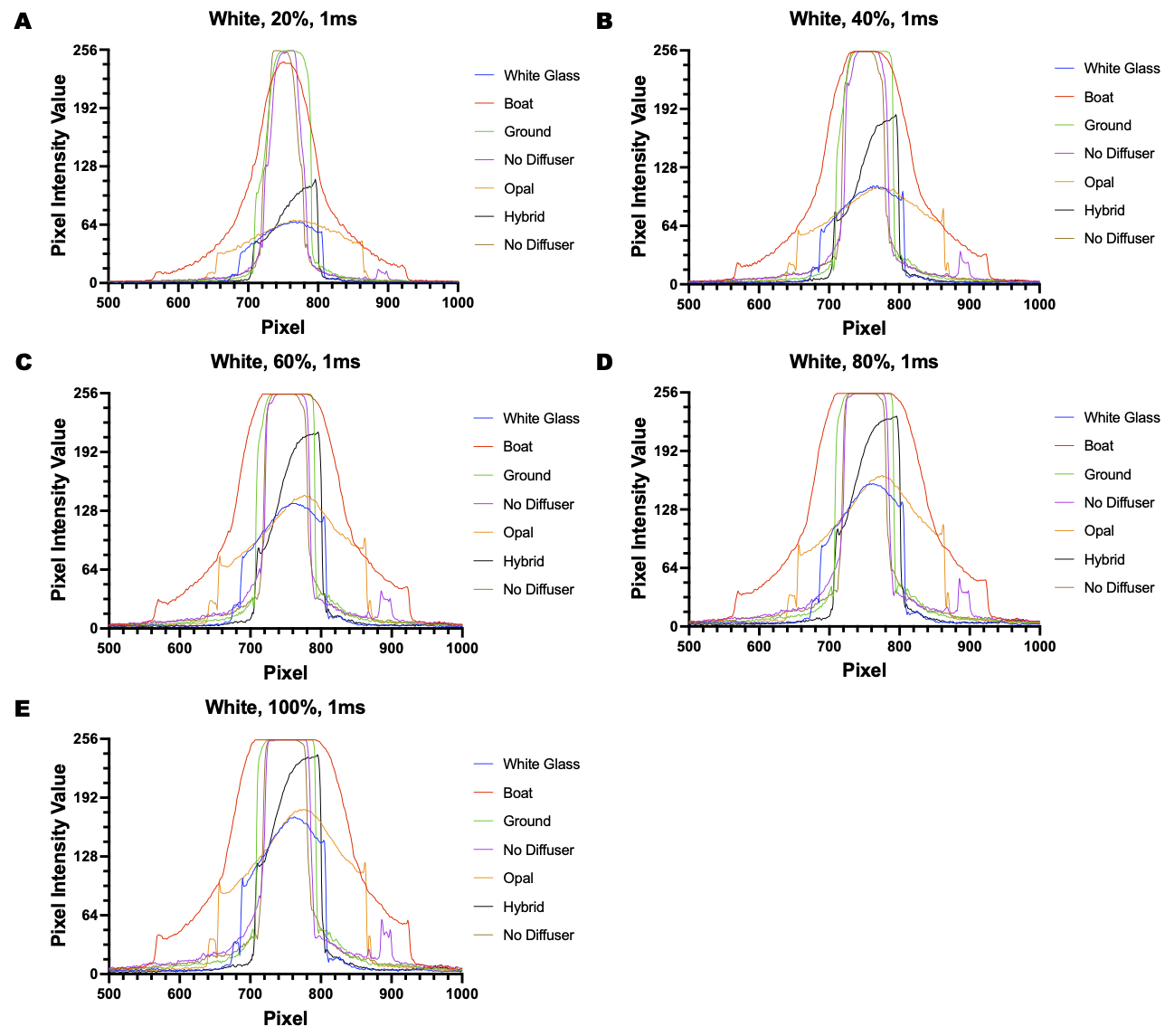

Supplement: S17 Fig — (A) White light at 20% intensity, (B) White light at 40% intensity, (C) White light at 60% intensity, (D) White light at 80% intensity, (E) White light at 100% intensity. (TIF) [file pone.0299875.s017.tif]

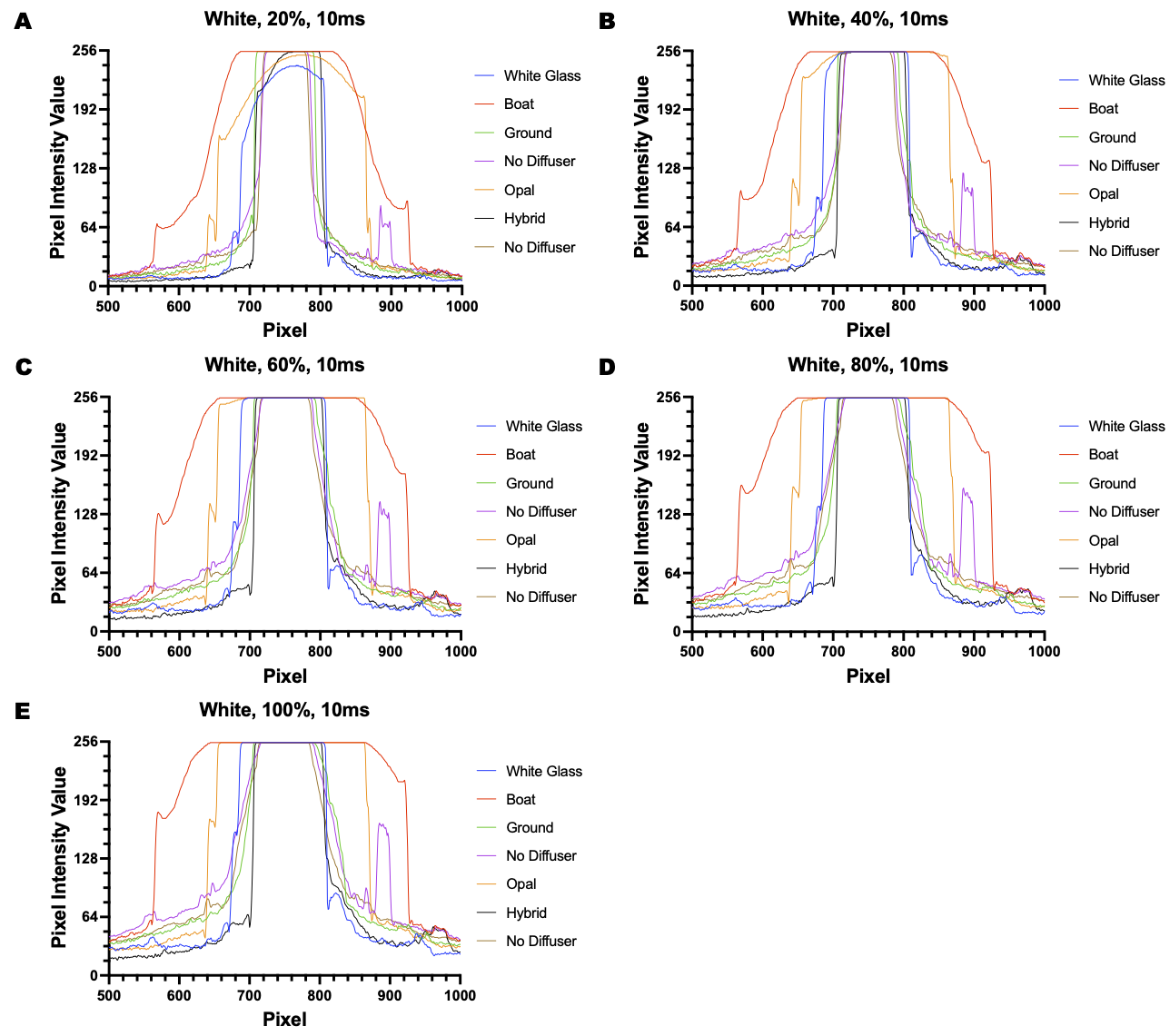

Supplement: S18 Fig — (A) White light at 20% intensity, (B) White light at 40% intensity, (C) White light at 60% intensity, (D) White light at 80% intensity, (E) White light at 100% intensity. (TIF) [file pone.0299875.s018.tif]

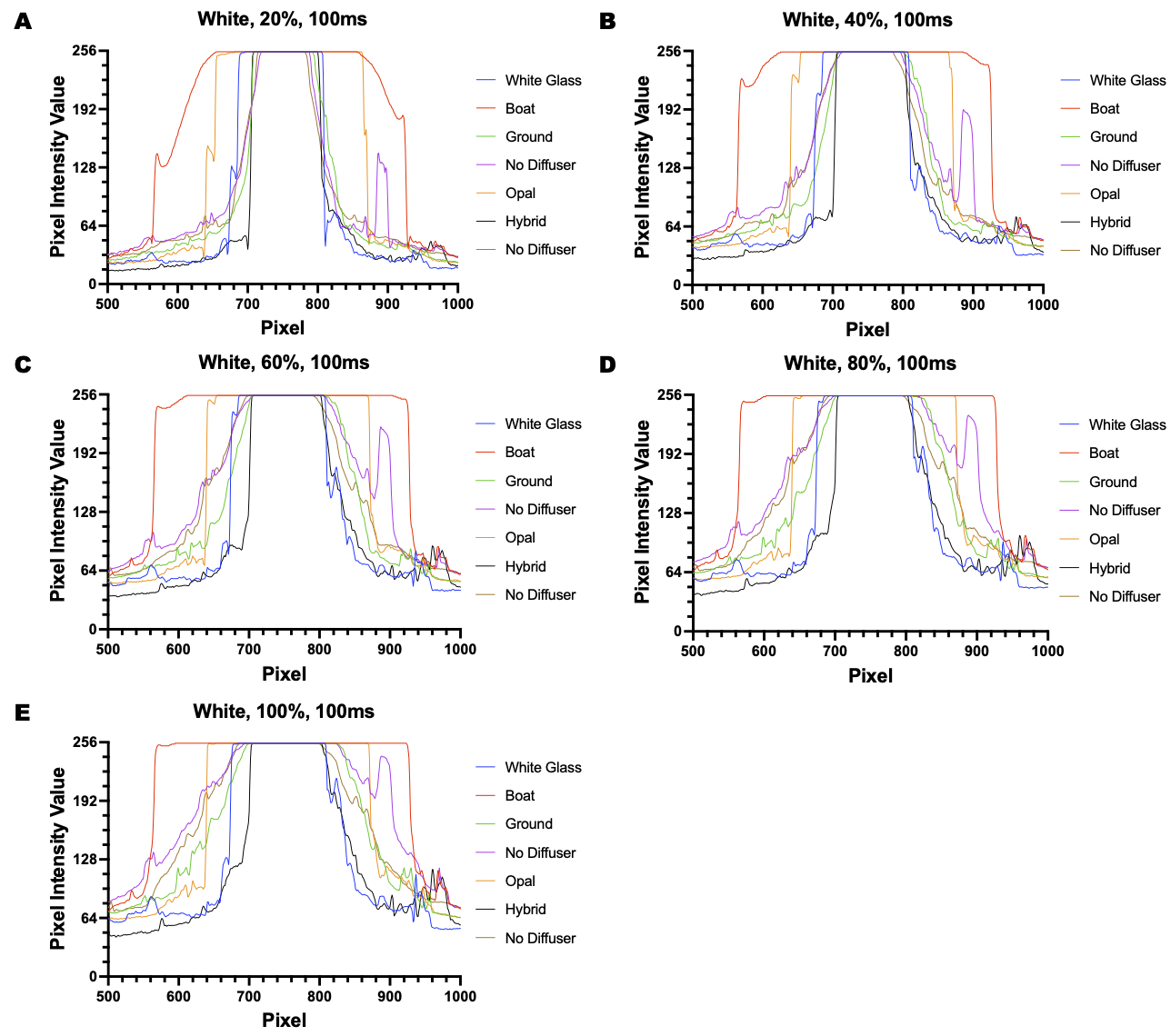

Supplement: S19 Fig — (A) White light at 20% intensity, (B) White light at 40% intensity, (C) White light at 60% intensity, (D) White light at 80% intensity, (E) White light at 100% intensity. (TIF) [file pone.0299875.s019.tif]

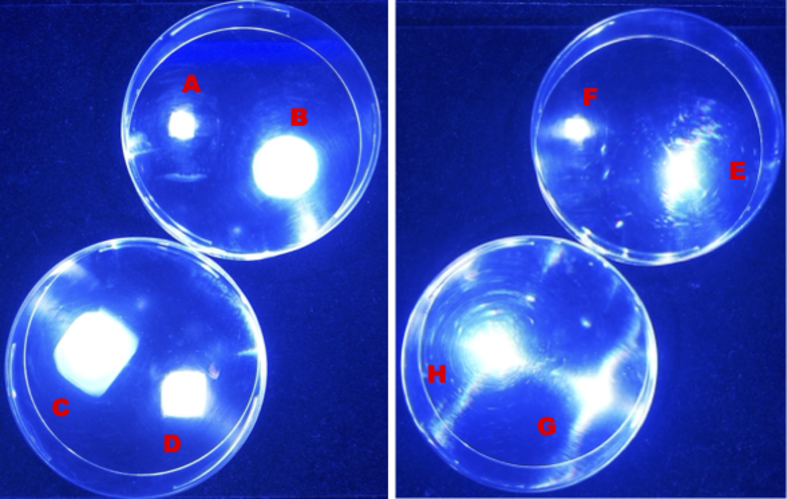

Supplement: S20 Fig — (A) White Diffuser Glass, (B) Weight Boat, (C) Weight Boat, (D) Diffuser Opal, (E) No diffuser, (F) Ground Glass, (G) Broadband Hybrid Diffuser, (H) No diffuser. (TIF) [file pone.0299875.s020.tif]
