# Supplementary material for: Multifunction fluorescence open source in vivo/in vitro imaging system (openIVIS)
Source: PLoS One. 2024 Mar 18;19(3):e0299875. doi: 10.1371/journal.pone.0299875 (PMC10947658; doi:10.1371/journal.pone.0299875)
Supplement: S1 Appendix — (DOCX) [file pone.0299875.s022.docx]

S1 Appendix System Operation

**Initial Configuration Steps**

There are several installation steps needed to get the Arducam IMX519 drivers installed on a Raspberry Pi. Adrucam has a Getting Started guide on their website(6). This guide provides instructions on how to install the IMX519 camera drivers and how to enable them in the boot config file. Adafruit has a guide on their website that provides and overview with instructions on how to control NeoPixel LEDs with Python on a Raspberry Pi(7).

**System Operation**

On the Raspberry Pi the python code is stored in /home/pi/Python. The open a terminal window either select the terminal icon on the menu task bar or via the start menu through “Accessories > Terminal”. Two methods to execute python scripts:

- In a terminal window type “cd /home/pi/Python”, then to execute a python file type “sudo python <filename>” in a terminal window where <filename> is the full python file name such as “fluorescence.py”
- For the standard scripts listed below, the “sudo python <filename>” was added as an alias in the “.bashrc” file in the /home/pi/ directory. This lets the user execute the commands from any directory.

**Experiment Operation**

For Fig 2, Fig 3, and Fig 8 the camera was operated using the command libcamera-still as follows: libcamera-still --autofocus --vflip --hflip -n --immediate -e png -o [filename]

For Fig 5, Fig6, Fig 7, Fig 9, and Fig 10 the camera was operated using the command libcamera-still as follows: libcamera-still --autofocus --vflip --hflip -n --immediate --raw 1 --shutter [XXX] -o [filename]

where the shutter value was looped through the values 1, 2, 4, 6, 8, 10, 20, 40, 60, 80, 100, 200, 400, 600, 800, 1000, 2000, 4000, 6000, 8000, 10000 to adjust the camera exposure time in milliseconds.

The LEDs were controlled using the NeoPixel library in python using the following commands:

pixels = neopixel.NeoPixel(board.D18, 8, brightness=1.0, auto_write=False, pixel_order=neopixel.RGB)

pixels = (desired color)

pixels.brightness = 1.0

pixels.show()

The desired colors are controlled by a three-integer value variable to set the RGB color. For blue LED (460 nm) excitation: pixels = (0,0,255), for green LED (520 nm) excitation: pixels = (0,255,0), for red LED (630 nm) excitation: pixels = (255,0,0), for white LED excitation: pixels = (255,255,255), for external excitation such as UV: pixels = (0,0,0). To change the LED power level the pixels.brightness was changed to a value between 0 – 1.0 based upon the desired output power.

**Camera Operation**

There are two methods to open a video stream from the camera. Note the “--autofocus” command syntax is dependent on version of the libcamera library installed and will vary slightly. The command “libcamera --help” will provide the details on the command syntax.

- Alias name “camera”. Type this command in any terminal window to execute the libcamera-script. This will open a video stream from the camera. To stop the video stream either close the video window or type “Ctrl+C” in the terminal window to terminate the command.
- Alias name “libcamera-still”. Type this command in any terminal window to execute the libcamera-script. There are many options with the script and user can type “libcamera-still --help” in a terminal window to see the various options.
  - Preview window: “libcamera-still --autofocus --vflip --hflip -t 0” this will open a video stream without a timeout, perform autofocusing, and perform a vertical and horizontal flip of the image to align the image with the back of the box at the top of the image.
  - Save image: “libcamera-still --autofocus --vflip --hflip --e png -o <filename>” this will perform autofocusing, perform a vertical and horizontal flip of the image to align the image with the back of the box at the top of the image, and save the image based on the user input <filename>

To edit python files, open the “File Manger” icon on the task bar. Change directory to “/home/pi/Python”. Right-click on the python file the user wants to edit and select “Geany”. This will open a built-in simplified IDE that allows the user to edit the python files.

**Standard Python files**

Python files with alias commands in the “*.bashrc*” file.

- **lightdimmer.py** : this one cycles through the LED colors with a 1-2 sec delay for each color. When the desired color is turned on the user needs to type “Ctrl+C” to cancel the python script. The LEDs will remain on with the last color. Alias name “dimmer”. Type this command in any terminal window to execute the python script.
- **fluorescence.py** : this one is the main imaging script for fluorescence measurements. The LED excitation color is controlled by the variable “colorLoop” on line 31 of the file. To add additional colors to the script, replace the number with the desired color, 0 = Red, 1 = Green, 2 = Blue, 3 = While, 4 = UV or external illumination. To execute multiple colors, the user needs to add a comma between colors, i.e. [0,1,2] to use Red, Green, and Blue excitation. The script will take both .png and .dng pictures for each exposure setting. The exposure settings are controlled by the variable *scale* on line 19 in the python file “saveImages.py”. The python script will ask the user for the experiment name. The experiment name will be used as the base string for each of the images with the LED color and exposure time appended to the image filename. Alias name “fluorescence”. Type this command in any terminal window to execute the python script. The python scripts save the images to “/home/pi/Pictures/<experiment name>” where <experiment name> is the user entered string when executing the python script.
