# Supplementary material for: Multifunction fluorescence open source in vivo/in vitro imaging system (openIVIS)
Source: PLoS One. 2024 Mar 18;19(3):e0299875. doi: 10.1371/journal.pone.0299875 (PMC10947658; doi:10.1371/journal.pone.0299875)
Supplement: S2 Appendix — (DOCX) [file pone.0299875.s023.docx]

S2 Appendix Alternative Single Board Computers

Alternative single board computers to the Raspberry Pi are discussed below. These are just several examples of the many alternatives to the Raspberry Pi that are available to developers and researchers. Ultimately, the choice of which board to use depends on your specific needs and requirements.

- ASUS Tinker Board: This is another single-board computer that is like the Raspberry Pi. It has a faster processor and more RAM, making it a good option for running more demanding applications(1).
- Nvidia Jetson Nano: The Jetson Nano is a powerful single-board computer designed for AI applications. It has a fast CPU and GPU and can handle advanced AI workloads. It is more expensive than the Raspberry Pi but offers better performance for certain applications(2).
- Libre Computer Board AML-S905X-CC (Le Potato): This is another single-board computer that is much like the Raspberry Pi. It has a similar processor and RAM and comes with a GPU. It has similar peripheral support making it an good alternative to the Raspberry Pi(3).
- BeagleBone Black: Like the Raspberry Pi, the BeagleBone Black is a small, single-board computer that can run Linux and can run a variety of applications. It has more built-in connectivity options and a faster processor than the Raspberry Pi(4).
- Odroid XU4: This is a powerful alternative to the Raspberry Pi that has a faster processor and more RAM. It is great for running more intensive applications such as media servers, gaming, and emulation(5).
