# Supplementary material for: Multifunction fluorescence open source in vivo/in vitro imaging system (openIVIS)
Source: PLoS One. 2024 Mar 18;19(3):e0299875. doi: 10.1371/journal.pone.0299875 (PMC10947658; doi:10.1371/journal.pone.0299875)
Supplement: S3 Appendix — (DOCX) [file pone.0299875.s024.docx]

S3 Appendix Temperature Effects

To determine the effect of the LEDs on the temperature inside the imaging box during long excitation durations a temperature logger (Elitech GSP-6) was used to record the temperature in ten second intervals while each of the eight LEDs were set to cycle through random colors for two hours. The temperature in the box increased three degrees over the course of the two hours with the temperature continuing to increase when the recording was stopped.
