# Supplementary material for: Multifunction fluorescence open source in vivo/in vitro imaging system (openIVIS)
Source: PLoS One. 2024 Mar 18;19(3):e0299875. doi: 10.1371/journal.pone.0299875 (PMC10947658; doi:10.1371/journal.pone.0299875)
Supplement: S4 Appendix — (DOCX) [file pone.0299875.s025.docx]

S4 Appendix Field of View Lenses

Images of the calibration chart taken with an Arducam OV2311 monochrome camera (UCTRONICS, Part Number B0381) with Arducam M12 Lens set (UCTRONICS, Part Number LK001). The camera was positioned 260 mm from the calibration chart for each picture.
