# Supplementary material for: Multifunction fluorescence open source in vivo/in vitro imaging system (openIVIS)
Source: PLoS One. 2024 Mar 18;19(3):e0299875. doi: 10.1371/journal.pone.0299875 (PMC10947658; doi:10.1371/journal.pone.0299875)
Supplement: S5 Appendix — (DOCX) [file pone.0299875.s026.docx]

S5 Appendix Light Diffusion Methods

The response of light diffusers on black acrylic were measured with varying light intensities and camera exposure times. The following diffusers were used Broadband Hybrid Diffuser (Edmund Optics, Part Number: 36611), Diffuser Opal (Edmund Optics, Part Number: 46167), Ground Glass (Edmund Optics, Part Number: 83083), White Diffuser Glass (Edmund Optics, Part Number: 34472), Weighing Boats (Thomas Scientific, Part Number: 22A00D595). Python was used to measure and plot the light intensity response across a 50x1500 pixel cross section through the center of the LED area. Standard petri dishes were used to determine the effect of the light diffusers on sample containers. White diffuser glass, opal diffuser, ground glass diffuser, and weight boats showed the best response.
