# Supplementary material for: Multifunction fluorescence open source in vivo/in vitro imaging system (openIVIS)
Source: PLoS One. 2024 Mar 18;19(3):e0299875. doi: 10.1371/journal.pone.0299875 (PMC10947658; doi:10.1371/journal.pone.0299875)

S1 Raw Images – DNA Gel Electrophoresis

A ThermoFisher Gene Ruler 1kb DNA ladder was stained with SYBR Safe and imaged. No experimental DNA outside of DNA ladder was used in imaging.

1^st^ Image – Used for Fig. 7A., 2^nd^ Image – Used for Fig 7B.

Lane Loading Order: 1, 2, 3, 4, 5, 6

Lane DNA Loading Weight: Lane 1 (10 µg); lane 2 (10 µg); Lane 3 (0 µg – no DNA, buffer only); Lane 4 (20 µg); Lane 5 (20 µg); Lane 6 (0 µg – no DNA, buffer only);


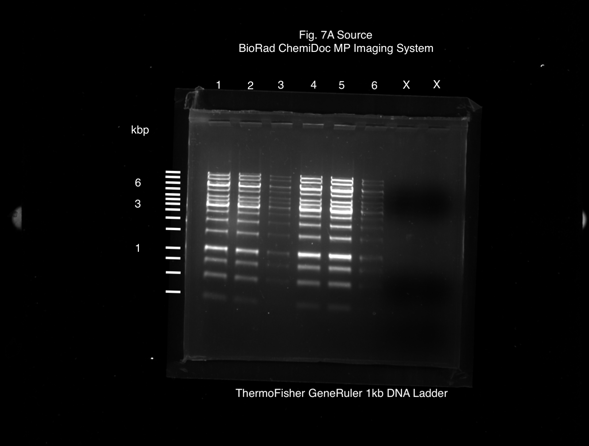


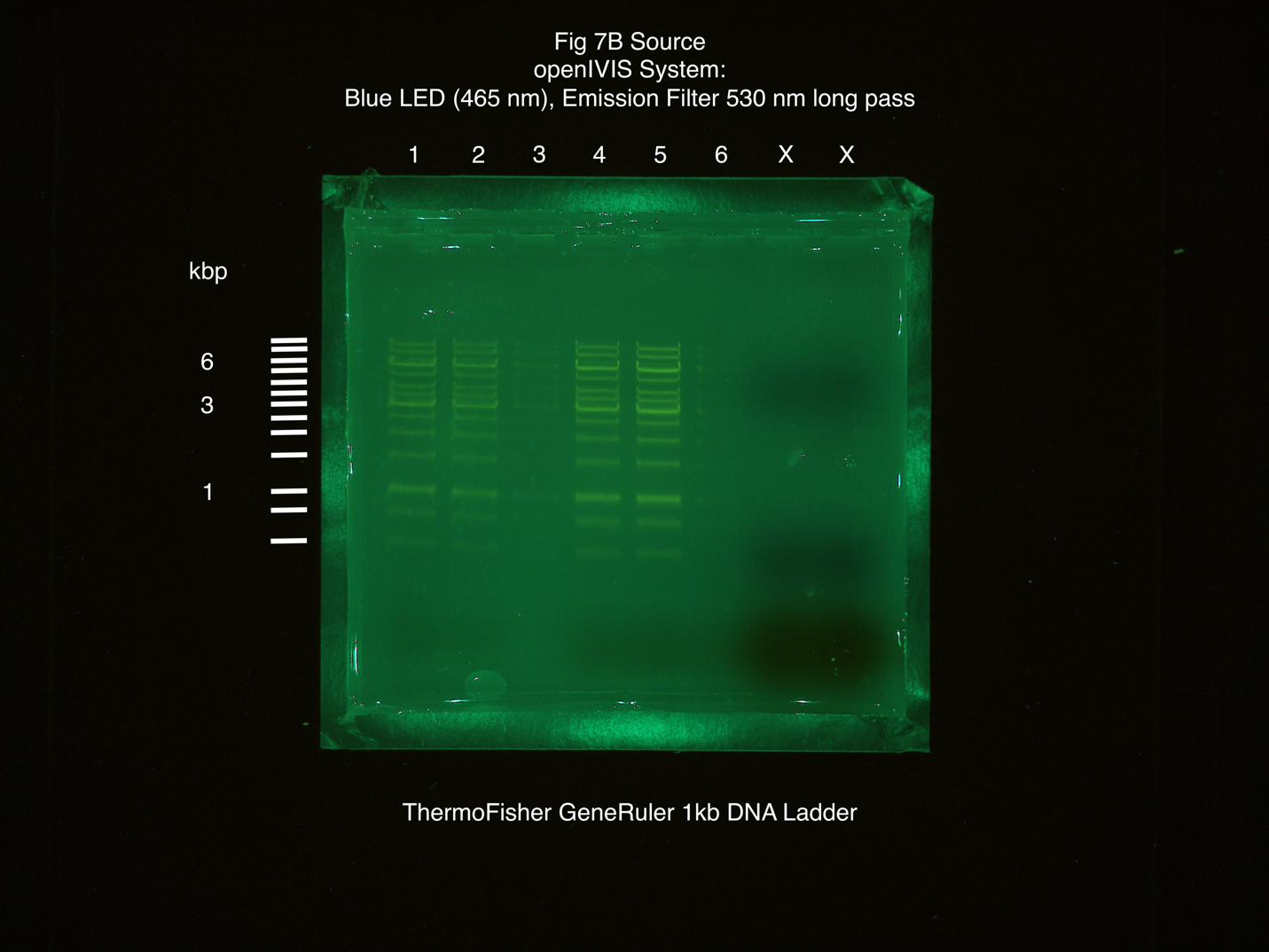

Supplement: S1 Raw images — (DOCX) [file pone.0299875.s027.docx]
